# Chromosomal deletions on 16p11.2 encompassing *SH2B1* are associated with accelerated metabolic disease

**DOI:** 10.1016/j.xcrm.2023.101155

**Published:** 2023-08-15

**Authors:** Ruth Hanssen, Chiara Auwerx, Maarja Jõeloo, Marie C. Sadler, Estonian Biobank Research Team, Elana Henning, Julia Keogh, Rebecca Bounds, Miriam Smith, Helen V. Firth, Zoltán Kutalik, I. Sadaf Farooqi, Alexandre Reymond, Katherine Lawler

**Affiliations:** 1 University of Cambridge Metabolic Research Laboratories, Wellcome-MRC Institute of Metabolic Science and NIHR Cambridge Biomedical Research Centre, Addenbrooke’s Hospital, Cambridge CB2 0QQ, UK; 2 Center for Integrative Genomics, University of Lausanne, 1015 Lausanne, Switzerland; 3 Department of Computational Biology, University of Lausanne, 1015 Lausanne, Switzerland; 4 Swiss Institute of Bioinformatics, 1015 Lausanne, Switzerland; 5 University Center for Primary Care and Public Health, 1010 Lausanne, Switzerland; 6 Institute of Molecular and Cell Biology, University of Tartu, 51010 Tartu, Estonia; 7 Estonian Genome Centre, Institute of Genomics, University of Tartu, 51010 Tartu, Estonia; 8 Department of Clinical Genetics, Cambridge University Hospitals NHS Foundation Trust & Wellcome Sanger Institute, Cambridge, UK

**Keywords:** *SH2B1*, 16p11.2, CNVs, precision medicine, obesity, type 2 diabetes, UK Biobank

## Abstract

New approaches are needed to treat people whose obesity and type 2 diabetes (T2D) are driven by specific mechanisms. We investigate a deletion on chromosome 16p11.2 (breakpoint 2–3 [BP2–3]) encompassing *SH2B1*, a mediator of leptin and insulin signaling. Phenome-wide association scans in the UK (N = 502,399) and Estonian (N = 208,360) biobanks show that deletion carriers have increased body mass index (BMI; p = 1.3 × 10^−10^) and increased rates of T2D. Compared with BMI-matched controls, deletion carriers have an earlier onset of T2D, with poorer glycemic control despite higher medication usage. Cystatin C, a biomarker of kidney function, is significantly elevated in deletion carriers, suggesting increased risk of renal impairment. In a Mendelian randomization study, decreased *SH2B1* expression increases T2D risk (p = 8.1 × 10^−6^). We conclude that people with 16p11.2 BP2–3 deletions have early, complex obesity and T2D and may benefit from therapies that enhance leptin and insulin signaling.

## Highlights

• Deletions on chromosome 16p11.2 are associated with early-onset obesity
• Deletion carriers have increased risk of early-onset type 2 diabetes
• Increased risk of metabolic disease may be mediated by decreased *SH2B1* expression

## Graphical abstract

**Figure:**
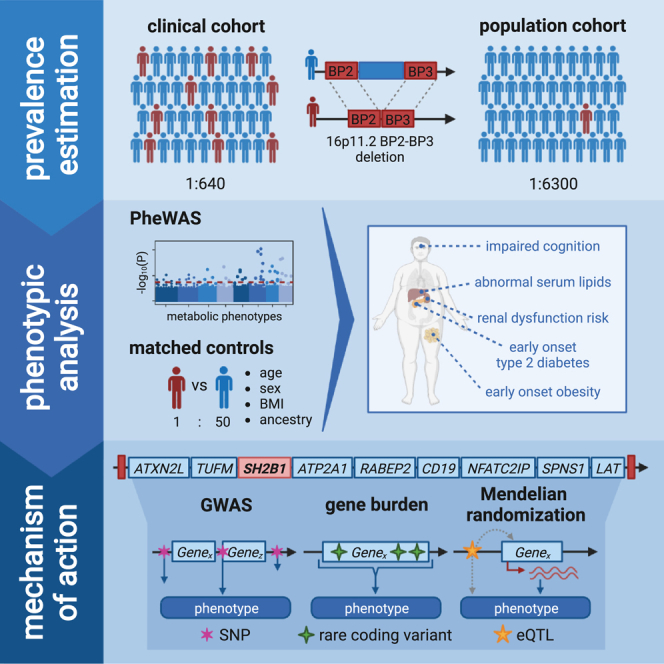

## Teaser

Leveraging the UK and Estonian Biobanks totalling over 700,000 individuals, Hanssen and Auwerx et al. demonstrate that people carrying a deletion on chromosome 16p11.2 containing the *SH2B1* gene have early-onset obesity and difficult-to-treat type 2 diabetes. Reduced *SH2B1* expression may explain these findings, providing opportunities for targeted treatment.

## Introduction

Obesity and type 2 diabetes (T2D) are highly prevalent, heterogeneous conditions associated with significant morbidity and mortality.^1^ The identification of subgroups of people whose metabolic disease is driven by shared pathogenic mechanisms can inform approaches to treatment. This is exemplified by monogenic forms of obesity due to penetrant rare variants affecting the development and/or function of the hypothalamic leptin-melanocortin pathway.^2^ Some of these disorders can now be treated with licensed therapies, such as recombinant leptin for congenital leptin deficiency or the MC4R agonist Setmelanotide for Leptin receptor (LEPR) (OMIM: 601007), POMC (OMIM: 176830), and PCSK1 (OMIM: 162150) deficiencies.^3^^,^^4^^,^^5^ *SH2B1* (Sarcoma homology 2 [SH2] B adaptor protein 1) (OMIM: 608937) acts as an intracellular adaptor that supports the assembly of proteins involved in leptin, insulin, and brain-derived neurotrophic factor (BDNF) signaling.^6^ *Sh2b1* knockout mice develop obesity, hyperglycemia, hepatic steatosis, and lipid accumulation in skeletal muscle.^7^^,^^8^^,^^9^ In humans, rare heterozygous loss-of-function mutations in *SH2B1* have been identified in children with hyperphagia, severe obesity, hyperinsulinemia, and maladaptive behavior.^10^^,^^11^^,^^12^ However, the trajectory of their metabolic disease in adulthood remains unclear.

**Figure 1:**
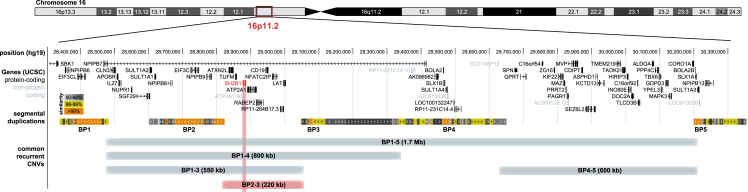
*SH2B1* encompassing 16p11.2 BP 2–3 deletions University of California Santa Cruz (UCSC) genome browser view of the 16p11.2 region (GRCh37/hg19). Upper track: exonic structure of genes in black (protein coding) or gray (non-protein coding). Middle track: segmental duplications forming the five breakpoint (BP) regions giving rise to recurrent copy-number variants (CNVs) at the 16p11.2 locus are colored according to the degree of similarity (light gray [90%] to orange [>99%]). Lower track: minimally deleted or duplicated region encompassed by the most common CNVs in the region. Recurrent CNVs are named after the BP regions that frame them (approximate size). Exact breakpoints occur at variable locations within the breakpoint region so that exact genomic coordinates and CNV length may differ between individuals. The 16p11.2 BP2–3 region, which represents the minimal and most common *SH2B1* encompassing deleted region, is highlighted in red.

Chromosome 16p11.2 contains five clusters of segmental duplications that increase the risk of recurrent copy-number changes at this locus through non-allelic homologous recombination^13^ (Figure 1). Copy-number variants (CNVs; duplications or deletions) with breakpoints (BPs) at these clusters have been reported in clinical^14^^,^^15^^,^^16^^,^^17^ and population-based cohorts.^18^^,^^19^^,^^20^^,^^21^ Rearrangement of the 600-kb proximal region (BP4–5) encompassing 33 genes (chr16:29.6–30.2 Mb; GRCh37) (OMIM: 611913) represents the most common deletion at the locus and has been associated with developmental delay, autism spectrum disorder (ASD), obesity, macrocephaly, and younger age at menarche.^18^^,^^19^^,^^20^^,^^21^^,^^22^^,^^23^^,^^24^^,^^25^ A smaller, 220-kb distal deletion (BP2–3; chr16:28.82–29.04 Mb; GRCh37) has been associated with early-onset obesity, macrocephaly, ASD and schizophrenia,^14^^,^^15^^,^^26^^,^^27^ and increased rate of obesity and T2D in population-based cohorts.^18^^,^^19^^,^^20^^,^^21^ The latter interval encompasses *SH2B1* and eight other protein-coding genes (Figure 1).

In this study, we characterized the clinical spectrum associated with the 16p11.2 BP2–3 deletion in adults from two population-based cohorts, the UK Biobank (UKBB) and Estonian Biobank (EstBB). Individuals recruited to population-based cohorts are typically older and healthier than individuals in clinically ascertained cohorts, allowing us to test hypotheses about the development, severity, and treatment of diseases and their complications.

## Results

### Prevalence of *SH2B1* encompassing 16p11.2 deletions

**Figure 2:**
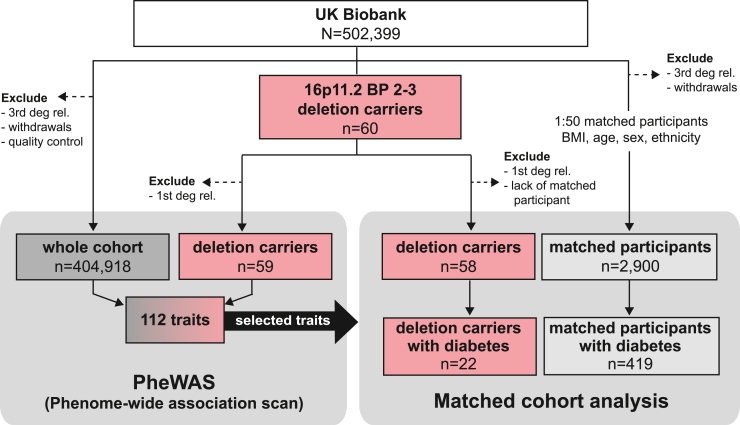
Study design Flow diagram (according to Consolidated Standards of Reporting Trails [CONSORT] principles) illustrating the detection of 16p11.2 BP2–3 deletion carriers in UKBB and the exclusion and inclusion criteria used to define the set of control individuals included in both the phenome-wide association scan (PheWAS) and matched cohort analysis. N represents the sample size of the whole UKBB, and n represents the subsets of individuals considered at various steps in the analysis. BMI, body mass index; deg. rel., degree relatives.

The UKBB is a cohort of 502,399 individuals (54% female) aged between 40 and 69 years at recruitment.^28^ To identify 16p11.2 BP2–3 deletion carriers (DELs), we used an automated CNV calling pipeline^19^ that feeds genotype microarray data to PennCNV^29^ and attributes a probabilistic quality score^30^ to each of the 272 deletions and 157 duplications identified across chr16:28.6–29.2 Mb (GRCh37). To avoid using an arbitrary quality score cutoff to select deletion carriers, fluorescent signal intensities (log R ratio [LRR]) and B-allele frequency (BAF) were manually reviewed in candidate deletion carriers, resulting in the detection of 60 unambiguous heterozygous deletion carriers with no other CNV in the 16p11.2 region. Of these, 51 (85%) had a quality score meeting the stringent cutoff (≤−0.5) previously used in genome-wide studies with no manual validation of CNV calls.^19^ After excluding one individual from a pair of first-degree relatives, we retained 59 unrelated deletion carriers for further analysis (Figure 2 and S1A; Table S1; STAR methods). These individuals comprised a similar proportion of males (DEL = 54%; UKBB = 46%; p_χ2_ = 0.257) and were slightly younger (mean_DEL_ = 54.5 years; mean_UKBB_ = 56.5 years; p_Wilcoxon_ = 0.046) than the whole UKBB cohort, with 52 (88.1%) individuals of self-reported and genetically estimated white British ancestry (Table 1; Figure S1B). In parallel and using a similar approach, we identified 19 unrelated deletion carriers in the EstBB (STAR Methods), a population-based cohort coupled to the national health system that encompasses 208,360 Estonians (65% females) aged between 18 and 103 years.^31^

**Table 1:** Characteristics of study participants

	PheWAS			Matched cohort analysis		
	Deletion carriers	UKBB	p	Deletion carriers	Matched controls	p
Sample size	59	404,918	–	58	2,900	–
Sex, male:female (%)	32:27 (54:46)	186,415:218,503 (46:54)	0.257	31:27 (53:47)	1,550:1,350 (53:47)	1
BMI (kg/m^2^)	31.67 ± 0.72	27.40 ± 0.01	1.3 × 10^−9^	31.66 ± 0.74	31.65 ± 0.10	0.991
Age (years)	54.54 ± 0.97	56.47 ± 0.01	0.046	54.71 ± 0.97	54.39 ± 1.35	0.752

Sample size, sex ratio (counts and percentage), and mean (± standard error [SE]) BMI and age for individuals studied in the PheWAS and matched cohort analysis. Deletion carriers are compared against non-carriers in the whole UKBB cohort (PheWAS) or BMI-matched controls (matched cohort analysis). Differences between the two groups were assessed through a chi-squared test (sex ratio) or Wilcoxon test (BMI and age) with the respective p value displayed.

We estimated the BP2–3 deletion frequency in UKBB as 1 in 6,868 (0.016%), which is concordant with previous estimates in UKBB^18^^,^^19^^,^^20^^,^^21^ and other population-based cohorts such as deCODE^32^ (Table S2). The slightly higher prevalence in the EstBB of 1 in 4,748 (0.021%) is likely due to differences in enrollment criteria. In comparison, estimates from clinical cohorts of children ascertained for various conditions, including developmental delay, was about 10-fold higher (1 in 642; 0.156%) (Table S2). Among considered cohorts, DECIPHER had the highest prevalence of deletion carriers, with estimates of 1 in 435 (0.230%). This online repository provides both genetic and phenotypic description of ∼45,700 patients with CNVs contributed by an international consortium of >200 academic clinical centers of genetic medicine and ≥1,600 clinical geneticists and diagnostic laboratory scientists.^33^ Specifically, 105 individuals carried the distal BP2–3 deletion; 24% of the 66 individuals on whom clinical information was available were reported to have obesity. Overall, our estimates are in line with results from a meta-analysis of 17 clinical and population-based cohorts that found a 16p11.2 BP2–3 deletion prevalence of 1 in 613 (0.163%) and 1 in 7,343 (0.014%) among individuals diagnosed with any or none of the 54 diseases investigated by the study, respectively.^34^

### Phenome-wide association scan in 16p11.2 BP2–3 deletion carriers in UKBB

**Figure 3:**
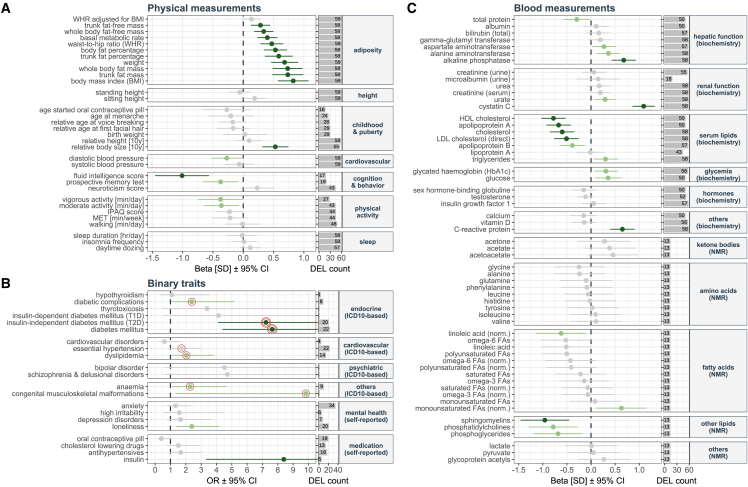
Phenome-wide association scan in carriers versus non-carriers of 16p11.2 BP2–3 deletions (A–C) Results of the PheWAS for 33 physical measurements (A), 21 binary traits (B), and 58 blood measurements (C) according to trait category (y axis). (A and C) Left panel, x axis shows the effect of the deletion (beta) on each trait in standard deviations (SDs) with error bars representing 95% confidence intervals (CIs). (B) Left panel, x axis shows the odds ratio (OR) with error bars representing the 95% CI. Upper range of the CI is truncated for some traits to facilitate visualization. Color indicates level of statistical significance: dark green (p ≤ 0.05/88 = 4.7 × 10^−4^), light green (p ≤ 0.05), and gray (non-significant). ICD-10-based diagnoses were assessed with a Cox proportional-hazards model and strictly (p ≤ 0.05/88 = 4.7 × 10^−4^) and nominally (p ≤ 0.05) significant associations between deletion carrier status and early onset of the disease are indicated by a double or single red circle surrounding the OR, respectively. The vertical dashed line represents a null effect size. Right panel, x axis indicates the number of deletion carriers (DEL, maximum n = 59) in whom the trait was measured (A and C) or the number of cases within deletion carriers for the considered trait (B). The PheWAS analysis included 404,918 non-carriers, with exact numbers of considered non-carriers reported in Tables S3, S4, S5, and S6.

To gain insights into the clinical characteristics of 16p11.2 BP2–3 deletion carriers, we designed a phenome-wide association scan (PheWAS) as a primary analysis, assessing 112 complex traits and hospital diagnosed diseases (International Classification of Diseases, 10^th^ Revision [ICD-10] codes) in 59 deletion carriers versus 404,977 unrelated UKBB non-carriers (Figure 3; Tables S3, S4, S5, and S6; STAR Methods). Estimating that the 112 traits correspond to 88 independent tests (STAR Methods), we identified 23 strictly significant associations (p ≤ 0.05/88 = 4.7 × 10^−4^) with deletion carrier status and 21 further nominally significant ones (p ≤ 0.05). As a sensitivity analysis to ensure that results were not affected by population stratification, we repeated the PheWAS on 52 deletion carriers versus 335,656 unrelated non-carriers of white British ancestry. Estimates obtained when considering only white unrelated British individuals were in high agreement with those of the whole cohort ($\rho_{Pearson}$ = 0.987; p < 2.2 × 10^−16^) supporting the robustness of our findings (Figure S2; Tables S3, S4, S5, and S6).

### 16p11.2 BP2–3 deletion is associated with increased adiposity

We found that 16p11.2 BP2–3 deletion carriers were significantly more likely to have a higher body mass index (BMI; β = 3.9 kg/m^2^; p = 1.3 × 10^−10^), weight (β = 10.8 kg; p = 2.0 × 10^−9^), whole-body fat mass (β = 7.0 kg; p = 5.9 × 10^−9^), and percentage fat mass (β = 4.5%; p = 5.9 × 10^−8^) (Figure 3A; Table S3). While waist-to-hip ratio appeared increased in deletion carriers (β = 0.47 standard deviation [SD]; p = 1.4 × 10^−6^), the effect disappeared upon correction for BMI (β = 0.13 SD; p = 0.109), suggesting no difference in fat distribution. Increased adiposity appeared in childhood, with 41.4% of deletion carriers self-reporting to be “plumper at age 10,” compared with 15.5% in the whole UKBB (p = 1.2 × 10^−6^). Neither childhood (p = 0.359) nor adult (p = 0.531) height was significantly associated with deletion carrier status. These results were replicated in the EstBB, where we found a significant increase in BMI (β = 3.7 kg/m^2^; p = 6.3 × 10^−4^) and weight (β = 10.0 kg; p = 2.2 × 10^−3^) among the 19 deletion carriers but no alteration in height (Table S3).

### 16p11.2 BP2–3 deletion carriers have early-onset T2D that is difficult to treat

**Figure 4:**
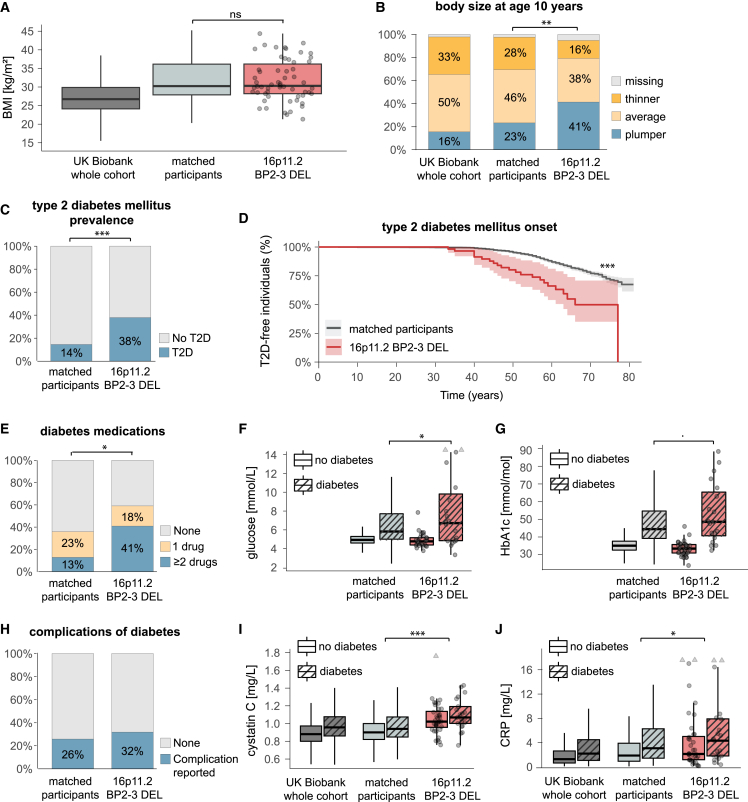
16p11.2 BP2–3 deletion carriers are at increased risk for early-onset T2D compared with BMI-matched non-carriers (A) BMI (kg/m^2^) of deletion carriers (16p11.2 BP2–3 DEL; red; n = 59) compared with UKBB whole cohort (dark gray; n = 403,280) and BMI-matched participants (light gray; n = 2,900). (B) Proportion (%) of individuals self-reporting their comparative body size at age 10 as plumper (blue), average (light yellow), or thinner (dark yellow); missing data (gray) among UKBB whole cohort (n = 396,450), matched participants (n = 2,900), and deletion carriers (16p11.2 BP2–3 DEL; n = 59); see Table S9A. (C) Prevalence (%) of T2D among deletion carriers (16p11.2 BP2–3 DEL; n = 58) and matched participants (n = 2,900); see Tables S9B and S9C. (D) Kaplan-Meier curves illustrating the proportion of T2D-free individuals (%) over time (years) among deletion carriers (16p11.2 BP2–3 DEL; red; n = 58) and matched participants (gray; n = 2,900). Shaded areas represent 95% CIs. (E) Proportion (%) of individuals taking no (gray), one (yellow), or several (blue) antidiabetic drugs among deletion carriers (16p11.2 BP2–3 DEL; n = 22) and matched participants with diabetes (n = 406); see Table S9D. (F and G) (F) Glucose (mmol/L) and (G) glycated hemoglobin (HbA1c) (mmol/mol) levels among deletion carriers (16p11.2 BP2–3 DEL; red; glucose n = 49; HbA1c n = 55) and matched participants (light gray; glucose n = 2,490; HbA1c n = 2,727) according to diabetic status. See Tables S9E and S9F. (H) Prevalence (%) of reported diabetic complications among deletion carriers (16p11.2 BP2–3 DEL; n = 22) and matched participants with diabetes (n = 406), see Table S9G. (I and J) Cystatin C (mg/L) levels according to diabetic status. UKBB whole cohort (dark gray) n = 385,797; matched participants (light gray) n = 2,698; deletion carriers (16p11.2 BP2–3 DEL; red) n = 58 (see Table S9G) and (J) C-reactive protein (CRP) (mg/L); UKBB whole cohort (dark gray) n = 384,965; matched participants (light gray) n = 2,691; deletion carriers (16p11.2 BP2–3 DEL; red) n = 58 (see Table S9H). Boxplot outliers are not shown for the whole cohort and matched participants. Data points depicted for deletion carriers (circles; triangles indicate values cropped at the maximum of the depicted range); ns, p > 0.1; ∗p < 0.05; ∗∗p < 0.01, ∗∗∗p < 0.001.

Our PheWAS indicated that 16p11.2 BP2–3 deletion carriers were at significantly increased risk for T2D (odds ratio [OR] = 7.2; p = 1.0 × 10^−11^) with considerably earlier onset of disease (hazards ratio [HR] = 6.1; p_Cox-PH_ = 8.4 × 10^−16^) and were more likely to receive insulin treatment (OR = 8.4; p = 6.9 × 10^−6^). They had nominally increased levels of glycated hemoglobin (HbA1c; β = 2.1 mmol/mol; p = 0.015) and random serum glucose (β = 0.4 mmol/L; p = 0.011) (Figures 3B and 3C; Tables S4, S5, and S6). The increased risk of T2D among deletion carriers was replicated in the EstBB (OR = 7.3; p = 2.5 × 10^−4^; Table S4). To test whether these results were driven by the increased adiposity observed in deletion carriers, we selected 50 controls (ctrl; unrelated non-carriers; i.e., UKBB participants who did not harbor the deletion) matched for BMI, age, sex, and self-reported ethnicity for 58 of the 59 deletion carriers (excluding one individual with <50 ethnicity-matched participants), amounting to a total of 2,900 matched non-carriers (Table 1; STAR Methods). Disease cases were defined using additional curation of self-reported clinical data, medication usage, biomarker levels, and physical measurements in addition to ICD-10 codes (Table S7). Even after matching for adult BMI (Figure 4A), deletion carriers more frequently reported to be plumper at age 10” (DEL = 41%; ctrl = 23%; p = 0.002; Figure 4B; Tables 2 and S9A), consistent with earlier onset of obesity. T2D prevalence was increased 2.7-fold (DEL = 38%; ctrl = 14%; p = 0.004; Figure 4C; Tables 2 and S9B) irrespective of body size at age 10 (all interactions DEL × comparative body size at age 10 with p > 0.27; Table S9B). Deletion carriers developed T2D at an earlier age than BMI-matched non-carriers (HR = 4.0; p_Cox-PH_ = 1.6 × 10^−7^; Figure 4D; Tables 2, S8, and S9C). A higher proportion of the 22 deletion carriers with T2D reported usage of antidiabetic drugs compared with the 419 matched non-carriers who had diabetes (DEL = 59%; ctrl = 36%; p = 0.033; Figure 4E; Tables 2 and S9D) and they were prescribed a larger number of medications (p = 0.022; Figure 4E; Tables 2 and S8). Despite higher antidiabetic medication usage, glycemic control measured by random serum glucose was worse in deletion carriers than in matched non-carriers with T2D (p_interaction T2D∗DEL_ = 0.006; *post hoc* analysis among cases, mean_DEL_ = 8.39 mmol/L; mean_ctrl_ = 6.97 mmol/L; p = 0.018; Figure 4F; Tables 2 and S9E). A similar trend was observed for HbA1c levels (p_interaction T2D∗DEL_ = 0.002; *post hoc* analysis, mean_DEL_ = 53.3 mmol/mol; mean_ctrl_ = 48.7 mmol/mol; p = 0.080; Figure 4G; Tables 2 and S9F).

**Table 2:** Metabolic characteristics of deletion carriers and BMI-matched controls

Category	Trait	Deletion carriers	Matched controls	p
Adiposity	prevalence of plumper at age 10 (%)	41.4	23.3	0.002
Glycemia	prevalence of T2D (%)	37.9	14.4	<2 × 10^−16^
	age of onset of T2D	51.1 ± 2.4	54.8 ± 0.5	1.6 × 10^−7^
	prevalence of diabetes treatment (%)	59.1	35.8	0.033
	number of antidiabetic drugs^a^	1.69 ± 0.13	1.37 ± 0.04	0.022
	glucose^a^ (mmol/L)	8.39 ± 1.18	6.97 ± 0.17	0.018
	HbA1c^a^ (mmol/mol)	53.3 ± 3.9	48.7 ± 0.7	0.080
	prevalence of diabetes with complications (%)	31.8	25.8	0.534
Renal function	cystatin C (mg/L)	1.077 ± 0.028	0.929 ± 0.003	6.0 × 10^−14^
Inflammation	C-reactive protein (mg/L)	4.84 ± 0.73	3.49 ± 0.09	0.015
Serum lipids	total cholesterol (mmol/L)	5.04 ± 0.14	5.62 ± 0.02	5.8 × 10^−5^
	triglycerides (mmol/L)	2.10 ± 0.17	1.97 ± 0.02	0.926
Cardiovascular system	prevalence of hypertension (%)	60.3	66.0	0.373
	diastolic blood pressure (mmHg)	79.8 ± 1.5	84.6 ± 1.9	2.8 × 10^−4^
	prevalence of cardiovascular diseases (%)	10.3	14.7	0.357

Descriptive statistics reporting the prevalence or mean value (± SE) for key metabolic phenotypes in deletion carriers and BMI-matched controls. Statistical significance of the difference between the two group is reported as a p value. Exact trait definitions, statistical tests, and further inferential statistics are described in Tables S7 and S8.

a Among people with documented diabetes.

### 16p11.2 BP2–3 deletion carriers have increased risk of renal impairment

Although the overall occurrence of known diabetic complications (retinopathy, kidney failure, polyneuropathy; Table S7) was comparable in 16p11.2 BP2–3 deletion carriers and matched controls (Figure 4H; Tables 2 and S9G), levels of cystatin C, an early biomarker of kidney dysfunction, were significantly elevated in deletion carriers compared with both the whole UKBB cohort (β = 0.19 mg/L; p = 2.0 × 10^−20^; Figure 3C; Table S6) and matched non-carriers (mean_DEL_ = 1.08 mg/L; mean_ctrl_ = 0.93 mg/L; p = 6.0 × 10^−14^; Figure 4I; Tables 2 and S9H) indicating that deletion carriers may be at increased risk of developing chronic kidney disease. Levels of C-reactive protein, a marker of chronic inflammation, were also increased in deletion carriers in both PheWAS (β = 2.8 mg/L; p = 5.1 × 10^−7^; Figure 3C; Table S6) and matched control analyses (mean_DEL_ = 4.84 mg/dL; mean_ctrl_ = 3.49 mg/dL; p = 0.015; Figure 4J; Tables 2 and S9I).

Hepatic steatosis is a common complication of obesity and T2D. Our PheWAS revealed increased serum levels of hepatic enzymes in deletion carriers (Figure 3C; Table S6) with significantly increased levels of alkaline phosphatase (ALP; β = 17.9 U/L; p = 1.2 × 10^−7^) and nominally increased levels of alanine (ALT; β = 5.1 U/L; p = 3.6 × 10^−3^) and aspartate (AST; β = 2.9 U/L; p = 0.034) aminotransferases. After controlling for alcohol consumption, diabetes, and lipid lowering drugs, only ALP (p = 1.9 × 10^−4^; Table S9J) and total bilirubin (p = 0.049; Table S9K) levels were increased in deletion carriers compared with BMI-matched non-carriers, while ALT, AST, and gamma-glutamyl transferase (GGT) levels did not differ between the groups (Tables S8 and S9L–S9N). Very few ICD-10-documented cases of non-alcoholic fatty liver disease are reported in UKBB; accordingly, no association with deletion carrier status could be detected (Tables S8 and S9O). Considering all liver diagnoses (K70–77), a higher proportion of deletion carriers was affected compared with non-carriers (p = 0.005; Table S9P). Specifically, deletion carriers had hepatic steatosis and cirrhosis diagnoses (mean age of onset = 64 years), possibly representing end-stage metabolic liver disease, which is often not accompanied by elevated liver enzymes.

**Figure 5:**
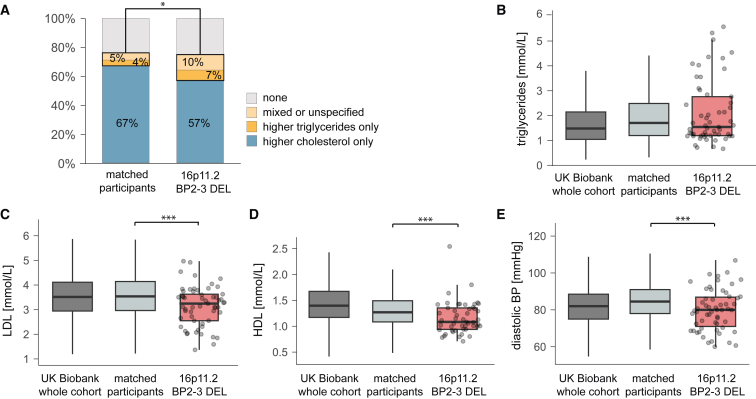
Cardiovascular risk factors in 16p11.2 BP2–3 deletion carriers compared with BMI-matched non-carriers (A) Proportion (%) of individuals with hypertriglyceridemia only (dark yellow), mixed or unspecified dyslipidemia (light yellow), hypercholesterolemia only (blue), or no dyslipidemia (gray) among deletion carriers (16p11.2 BP2–3 DEL; n = 58) and BMI-matched participants (n = 2,900); see Table S9Q. Star indicates significance for the comparison of hypertriglyceridemia and mixed/unspecified dyslipidemia between 16p11.2 BP2–3 DEL and matched participants. (B) Serum triglycerides (mmol/L). UKBB whole cohort (dark gray) n = 385,495; matched participants (light gray) n = 2,695; deletion carriers (16p11.2 BP2–3 DEL; red) n = 57. (C) LDL-cholesterol levels (mmol/L). UKBB whole cohort (dark gray) n = 385,079; matched participants (light gray) n = 2,692; deletion carriers (16p11.2 BP2–3 DEL; red) n = 57 (see Table S9T). (D) HDL-cholesterol (mmol/L). UKBB whole cohort (dark gray) n = 353,195; matched participants (light gray) n = 2,496; deletion carriers (16p11.2 BP2–3 DEL; red) n = 49 (see Table S9W). (E) Diastolic blood pressure (BP) (mmHg) levels. UKBB whole cohort (dark gray) n = 404,478; matched participants (light gray); deletion carriers (16p11.2 BP2–3 DEL; red) n = 58 (see Table S9Z). Boxplot outliers are not shown for the whole cohort and matched participants. Data points depicted for deletion carriers (circles). ∗p < 0.05; ∗∗∗p < 0.001.

To study dyslipidemia in the matched cohort setting, we considered ICD-10-coded and self-reported dyslipidemia, as well as blood-panel-derived cases (Table S7). Prevalence of dyslipidemia in deletion carriers was not increased after accounting for BMI (Figure 5A; Table S9Q). However, the proportion of individuals with hypertriglyceridemia only or mixed dyslipidemia was increased in deletion carriers (DEL = 17%; ctrl = 9%, p = 0.029; Figure 5A; Table S9R), findings that may be explained by their suboptimal glycemic control. We observed that triglyceride levels were comparable between deletion carriers and matched non-carriers (Figure 5B; Tables 2 and S8), while low-density lipoprotein (LDL)-cholesterol, total cholesterol, and apolipoproteins A and B levels were significantly decreased in deletion carriers compared with the whole UKBB cohort (Figure 3C; Table S6) and matched non-carriers (all p < 0.003; Tables S9S–S9V; Figure 5C). High-density lipoprotein (HDL)-cholesterol levels followed the same trend and were decreased compared with both the UKBB cohort (β = −1.13 mmol/L; p = 9.2 × 10^−10^; Figure 3C; Table S6) and matched non-carriers (mean_DEL_ = 1.17 mmol/L; mean_ctrl_ = 1.32 mmol/L; p = 4.8 × 10^−9^; Figure 5D; Table S9W). There was no increase in the use of cholesterol-lowering drugs in deletion carriers in the PheWAS or matched cohort analysis (Figure 3B; Tables S5 and S9S–S9V).

Nuclear magnetic resonance (NMR) spectroscopy revealed that serum levels of linoleic acid, sphingomyelins, phosphatidylcholines, and phosphoglycerines were significantly reduced in deletion carriers compared with the UKBB cohort (Figure 3C; Table S6) despite availability of these measurements in only 13 deletion carriers. Cross-sectional and longitudinal studies have shown that higher levels of linoleic acid are associated with decreased incidence of T2D,^35^ which aligns with deletion carriers having both lower levels of the metabolite and increased incidence of T2D. Furthermore, these results are concordant with a previous study of patients with obesity with T2D who were found to have lower levels of sphingomyelin, an abundant sphingolipid involved in ceramide metabolism, compared with people with obesity without T2D.^36^

Although the prevalence and age of onset of hypertension were not significantly different between deletion carriers and matched non-carriers (Tables S8 and S9X), diastolic blood pressure was lower in deletion carriers compared with the whole UKBB cohort (β = −2.8 mmHg; p = 0.033; Figure 3A; Table S3). This trend was preserved in comparison to BMI-matched non-carriers, irrespective of the use of antihypertensive medication (mean_DEL_ = 79.8 mmHg; mean_ctrl_ = 84.6 mmHg; p = 2.8 × 10^−4^; Figure 5E; Tables S8 and S9Y–S9Z). Neither the PheWAS (Figure 3B; Table S4) nor the matched participant analysis (Table 2; Tables S8 and S9AA) found deletion carriers to be at increased risk for cardiovascular disease.

### 16p11.2 BP2–3 deletions are associated with additional non-metabolic phenotypes

ASD and developmental delay have previously been associated with 16p11.2 BP2–3 deletions.^26^ However, UKBB individuals present with a lower disease burden compared with the general UK population^37^ and ASD prevalence in UKBB is about 0.05%, compared with a recent estimate of 1.76% across 7 million English school children.^38^ Accordingly, none of the UKBB deletion carriers were diagnosed with ASD, suggesting that carriers from the general population are at the milder end of the phenotypic range, paralleling what has been shown for other CNVs.^39^^,^^40^ Self-reported behaviors can indicate features that lie at the mild end of the clinical spectrum. The PheWAS indicated that deletion carriers report higher rates of loneliness (OR = 2.4; p = 0.002; Figure 3B; Table S5), a trend maintained in the matched cohort analysis (DEL = 34%; ctrl = 21%; p = 0.036; Figure S3A; Table S9AB). We found no significant differences in prevalence of anxiety, irritability, or depressive disorders in deletion carriers compared with the whole UKBB cohort and matched non-carriers (Figure 3B; Tables S5 and S9AC–S9AE), but cognitive ability seemed to be impaired among deletion carriers, who performed worse on both fluid intelligence (p_PheWAS_ = 8.6 × 10^−6^; Figure 3A; Table S3; p_matched control_ = 8.1 × 10^−4^; Figure S3B; Table S9AF) and prospective memory tests (p_PheWAS_ = 0.013; Figure 3A; Table S3; p_matched control_ = 0.047; Table S9AG).

The PheWAS also revealed a nominally significant increased risk (OR = 2.3; p = 0.024) and earlier onset (HR = 2.1; p = 0.022) of anemia among 16p11.2 BP2–3 deletion carriers (Figure 3B; Table S4). Similarly, anemia was more prevalent in deletion carriers than in matched non-carriers (Table S9AH; Figure S3C). Hemoglobin, hematocrit, mean corpuscular hemoglobin and volume, and reticulocyte count were all higher in deletion carriers compared with matched non-carriers (Tables S8 and S9AI–AN).

### Mechanism of action of 16p11.2 BP2–3 deletions

We investigated whether haploinsufficiency of the nine genes mapping to the 16p11.2 BP2–3 interval could corroborate the increased BMI and T2D risk observed in deletion carriers. We explored rare variant gene burden association summary statistics for BMI and T2D performed in 454,787 whole exomes of the UKBB using different masks on variant function and minor allele frequency (MAF).^41^ Rare (MAF ≤ 0.001%) predicted loss-of-function (pLoF) variants in *NFATC2IP* were associated with increased BMI at nominal significance (β = 0.32; p = 0.012). Interestingly, while the burden of pLoF and predicted deleterious missense variants in *ATXN2L* (OR = 0.76; p = 0.011) and *SPNS1* (OR = 0.89; p = 0.032) nominally decreased T2D risk, the singleton burden in *SH2B1* nominally increased it (OR = 2.5; p = 0.028) (Table S10). Similarly, we investigated whether gene burden test results supported the unusual pattern in serum lipid levels observed among deletion carriers, characterized by a reduction in both LDL and HDL levels, compared with BMI-matched controls. Concordantly, singleton loss-of-function burden in *SH2B1* decreased both total cholesterol (β = −0.63; p = 0.002) and LDL (β = −0.58; p = 0.005) levels, and while rare variants (MAF ≤0.01%) in *SH2B1* also decreased HDL levels (β = −0.19; p = 0.022), more significant HDL-decreasing (*ATP2A1*, p = 0.002; *LAT*, p = 0.010) and -increasing (*RABEP2*, p = 0.013) effects were observed for other genes in the region (Table S10).

Next, we assessed whether common single-nucleotide variants in the 16p11.2 BP2–3 interval ±50 kb were associated with traits affected by the deletion. We retrieved 287 association signals (p < 9 × 10^−6^) from the genome-wide association study (GWAS) catalog^42^ (Table S11; STAR Methods), including signals related to adiposity (n = 95), cognitive function (n = 38), anemia (n = 17), serum lipid levels (n = 5), renal function (n = 4), diabetes (n = 3), physical activity (n = 2), and hepatic function (n = 2) (Figure S4A). Other signals were related to traits not assessed by our PheWAS, e.g., related to the reward system, immunity, autoimmunity or brain morphology, and represent interesting leads for future investigation. About half of the reported signals mapped to *ATXN2L* (n = 85) and *SH2B1* (n = 66), the two genes in the region under the strongest evolutionary constraint according to GnomAD (probability of LoF intolerance [pLi] = 1; LoF observed over expected upper bound fraction [LOEUF] < 0.23).^43^ Focusing on the 95 adiposity-related signals, 30 and 20 were reported to map to *SH2B1* and *ATP2A1*/*SH2B1*, respectively. However, the low recombination rate over the region prevents accurate fine mapping of GWAS signals (Figure S4A).

To gain further resolution, we used transcriptome-wide Mendelian randomization (TWMR),^44^ a causal inference approach that aims at identifying statistical causal links between changes in gene expression levels and an outcome, here T2D risk (Figure S4B). We could evaluate the causal impact of expression changes on T2D risk for six out of the nine 16p11.2 BP2–3 genes that had at least one eQTL (expression quantitative trait locus) variant in blood^45^ (Figure S4C; Table S12; STAR Methods). Among the four genes with a significant TWMR effect (p ≤ 0.05/9 = 5.6 × 10^−3^), only *SH2B1* had a directionally concordant effect (α = −0.23; p = 8.1 × 10^−6^) with the one observed in our CNV association study, i.e., increased *SH2B1* expression decreased T2D risk, which is compatible with the deletion reducing the gene’s expression and increasing T2D risk. While blood offers the largest eQTL datasets, this tissue is unlikely to mediate metabolic phenotypes. We repeated this analysis using smaller-sized tissue-specific eQTLs from the Genotype-Tissue Expression (GTEx) project^46^ available for six out of nine genes (Table S13; STAR Methods). Results were consistent across tissues, with increased expression of *ATP2A1*, *NFATC2IP*, *SPNS1*, and *TUFM* increasing T2D risk, and increased expression of *SH2B1* and *ATXN2L* decreasing risk for T2D, even if for the latter the effect was only found in whole blood. These results align with results obtained from the eQTLGen dataset and highlight *SH2B1* as the best candidate gene for the increased T2D risk observed in deletion carriers, involving brain, adipose tissue, and muscle as plausible effector tissues. One caveat is that all but one TWMR estimate for *SH2B1* relies on a single eQTL. Seeking further evidence that changes in *SH2B1* expression affect T2D, we performed colocalization analysis^47^ between the T2D GWAS signal and expression levels of the four genes with a significant TWMR effect but could not find any evidence of a shared causal variant (posterior probability of signal colocalisation [PP_H4] <0.387) (Table S14; STAR Methods).

## Discussion

We show that people who are heterozygous carriers of 16p11.2 BP2–3 deletions have a higher rate of obesity, which is typically earlier in onset and associated with an accelerated form of metabolic disease characterized by early and more difficult-to-treat T2D. Experimental studies in animals will be needed to test whether disruption of *SH2B1* and/or other genes in this locus cause accelerated chronic liver disease, as suggested by our findings.

These findings have direct clinical relevance as current clinical guidelines recommend that people with severe, early-onset obesity (≤5 years) should be offered genetic testing.^48^ While targeted gene panels or whole-exome sequencing are the most frequently offered investigations, they are often blind to chromosomal rearrangements unless the diagnosis pipeline uses depth-of-coverage maps to identify deleted exons and CNVs. The latter approach, or alternatively array CGH (comparative genomic hybridization) or MLPA (multiplex ligation-dependent probe amplification), should be considered to detect 16p11.2 BP2–3 deletions in children and young adults presenting with obesity and features of insulin resistance and/or early or difficult-to-treat T2D. Deletions involving 16p11.2 BP2–3 may be identified by a range of physicians who organize genetic testing to investigate developmental delay and ASD. It is important that diagnosed individuals are also reviewed by endocrinologists so that weight loss therapies, insulin sensitizers, and other glucose-lowering agents can be started at a young age to limit the impact of poor glycemic control and prevent the complications of accelerated metabolic disease.

To examine potential mechanisms underlying the observed associations, we investigated the individual contribution of the nine genes in the 16p11.2 BP2–3 interval to associated phenotypes. Among these, four genes are associated with autosomal recessive disorders: *ATP2A1* with Brody myopathy (OMIM: 601003), *TUFM* with combined oxidative phosphorylation deficiency 4 (OMIM: 610678), and both *CD19* and *LAT* with common variable immunodeficiency 3 (OMIM: 613493) and immunodeficiency 52 (OMIM: 617514), respectively. Heterozygosity of the latter was also proposed to drive increased head circumference in deletion carriers.^49^ Furthermore, experiments in mice have shown that homozygous ablation of *Atxn2l* causes lethal *in utero* brain lamination defects.^50^ The International Mouse Phenotyping Consortium found that heterozygous deletion of *Spns1* leads to increase in both total body fat and lean body mass,^51^ and a recent study demonstrated the role of the encoded protein in lysosomal lysophospholipid efflux,^52^ warranting further investigation to determine whether the gene is involved in the reduced levels of phosphatidylcholines, phosphoglycerides, and sphingomyelins observed in deletion carriers. As people carrying rare dominant mutations in *SH2B1* and *Sh2b1* knockout mice have obesity and insulin resistance,^7^^,^^8^^,^^9^^,^^12^ *SH2B1* appears to be the most likely candidate gene for the metabolic phenotype observed in 16p11.2 BP2–3 deletion carriers. These results are supported by our tissue-specific TWMR analysis, which suggests the importance of *SH2B1* expression in the brain, adipose tissue, and muscle in mediating T2D susceptibility. However, it remains unclear whether epistatic interactions resulting from the deletion of multiple genes could contribute to phenotypes unique to 16p11.2 BP2–3 deletion carriers.

Our clinical description of a large cohort of adult 16p11.2 BP2–3 deletion carriers indicates phenotypes that overlap with previous reports of people with SH2B1 deficiency. For instance, leptin couples changes in weight to changes in blood pressure so that mice and humans lacking leptin or its receptor have low blood pressures, despite severe obesity,^53^ in line with the reduced diastolic blood pressure seen in deletion carriers compared with BMI-matched non-carriers. Furthermore, studies in mice and humans have suggested that leptin stimulates hepatic triglyceride export via the brain-vagus nerve-liver axis,^54^ which may explain the increased levels of hepatic biomarkers and lower lipid levels seen in deletion carriers. The lack of reduction of triglyceride levels in deletion carriers may be explained by the poorer glycemic control seen in deletion carriers. In the brain, SH2B1 mediates BDNF signaling.^55^ In humans, loss of function of BDNF and its receptor TrkB, as well as SH2B1 deficiency, have been associated with speech and language delay, behavioral abnormalities, and memory impairment,^10^^,^^55^^,^^56^ features overlapping the behavioral and cognitive phenotypes seen in deletion carriers. Finally, SH2B1 acts as a negative regulator of erythropoietin receptor-mediated signaling,^57^ which may in part explain the increased blood count values seen in deletion carriers. These findings require further investigation to delineate the underlying mechanisms.

### Limitations of the study

Our study has several limitations. First, population-based cohorts suffer from ascertainment bias as individuals with a high disease burden, such as 16p.11.2 BP2–3 deletion carriers, are less likely to volunteer for research studies. This decreases the case number of an already rare genetic alteration, limiting the statistical power to dissect the health consequences of the 16p11.2 BP2–3 deletion. Power is further limited as carriers present in the cohort have milder clinical phenotypes. A second limitation is the lack of advanced clinical measurements of insulin sensitivity, or the inability to recall individuals based on their genotype to perform additional investigations (e.g., hyperinsulinemic euglycemic clamps), which would allow a more detailed understanding of the metabolic consequences of the deletion. Finally, our attempt at pinpointing individual genes responsible for the phenotypic associations is limited by several factors, including (1) the lack of sufficiently variable CNV breakpoints in the region,^19^ (2) the low frequency of pLoF variants in evolutionary constrained genes in the region, (3) the low recombination rate that hinders fine-mapping of common variant association signals, and (4) the lack of sufficient eQTLs to robustly instrument TWMR analyses. The latter is particularly relevant as it makes our analysis susceptible to violation of Mendelian randomization (MR) assumptions. Indeed, while colocalization did not unambiguously favor any scenario, highest support was given to H_3_ (PP_H3: 0.60–0.76). This possibly indicates that different variants underly the change in gene expression and T2D risk, violating the second MR assumption through linkage-disequilibrium-induced horizontal pleiotropy. However, the high probability of H_3_ may only reflect that there are multiple underlying signals for both traits, violating the assumption of the colocalization method, hence it is inconclusive regarding the MR assumption violation. Although there are substantial experimental data to support the role of SH2B1 in mediating the phenotypes of obesity, T2D, and fatty liver disease, further studies are needed to examine the potential phenotypic contribution of other coding genes and noncoding RNAs affected by the 16p11.2 BP2–3 deletion. In the future, availability of large, longitudinal clinical and population cohorts with detailed phenotypic data should mitigate these hurdles.

In conclusion, 16p11.2 BP2–3 deletion carriers have a subtype of obesity that is characterized by early onset of metabolic complications including T2D. People with this disorder should be considered for early intervention with weight-loss therapies. The results of ongoing phase 3 clinical trials of Setmelanotide, an MC4R agonist in genetic obesity syndromes (ClinicalTrials.gov: NCT05093634) will provide critical information as to whether people with pathogenic mutations in *SH2B1* and with 16p11.2 BP2–3 deletions may benefit from treatment with drugs that improve signaling through the leptin-melanocortin pathway.^4^ Indeed, if the clinical trial demonstrates that 16p11.2 BP2–3 deletion carriers lose a significant amount of weight, this will provide orthogonal evidence of the contribution of SH2B1 to the obesity of deletion carriers, as people with common obesity are unlikely to respond to MC4R agonism. Collectively, these findings highlight the growing importance of mechanism-based approaches to the treatment of patients with subtypes of severe obesity.

## STAR★Methods

### Key resources table

**Table:** 

REAGENT or RESOURCE	SOURCE	IDENTIFIER
**Deposited data**		

UK Biobank, application numbers 16389 and 53821	Bycroft et al.^28^	https://www.ukbiobank.ac.uk/
Estonian biobank in the data freeze 2022v01 (2022-04-12); release application 6–7/GI/2018 [2023/01/18]	Leitsalu et al.^31^	https://www.eithealth-scandinavia.eu/biobanks/the-estonian-biobank/
DECIPHER	Firth et al.^33^	https://www.deciphergenomics.org
HapMap (Phase II) recombination map lifted over to GRCh37/hg19	International HapMap Consortium et al.^58^	https://github.com/odelaneau/shapeit5/tree/main/maps/genetic_maps.b37.tar.gz
NHGRI-EBI GWAS Catalog	Sollis et al.^42^	https://www.ebi.ac.uk/gwas/
Gene burden tests from whole exome sequencing data in the UK Biobank; Deposited on the GWAS Catalog.	Backman et al.^41^	N/A
Type 2 diabetes GWAS; Deposited on the GWAS Catalog.	Mahajan et al.^59^	GWAS Catalog identifier: GCST007517
eQTLGen Consortium (Phase I)	Võsa et al.^45^	https://www.eqtlgen.org/phase1.html
The Genotype-Tissue Expression (GTEx) project (V8)	GTEx Consortium^46^	https://gtexportal.org/home/

**Software and algorithms**		

PennCNV	Wang et al.^29^	https://penncnv.openbioinformatics.org
CNV quality score pipeline	Macé et al.^21^	http://goo.gl/T6yuFM
UCSC LiftOver	UCSC Genome Browser	https://genome.ucsc.edu/cgi-bin/hgLiftOver
smrivw (v1.1)	Sadler et al.^60^	https://github.com/masadler/smrivw
R v3.6.1 and v4.1.1 (statistical analyses) and v4.1.3 (graphs)	R Foundation for Statistical Computing	https://www.r-project.org/

**Other**		

UK Biobank CNV calls	Auwerx et al.^19^	N/A

### Resource availability

#### Lead contact

Further information and requests for resources should be directed to and will be fulfilled by the lead contact, Sadaf Farooqi (isf20@cam.ac.uk).

#### Materials availability

This study did not generate any unique reagents.

### Experimental model and study participant details

#### UK biobank

This research was conducted using the UK Biobank resource under application numbers 16389 and 53821. The UK Biobank (UKBB) is a voluntary-based cohort of 502,399 individuals (54% females) from the general UK population that were recruited at age 40–69 years, signing a broad informed consent form for the usage of their data.^28^

#### Phenome-wide association scan

Primary phenome-wide association scan (PheWAS) was carried out on a set of 404,977 individuals of mixed ancestry retained after filtering out i) related samples (≤3^rd^ degree, preference given to 16p11.2 break point (BP) 2-3 deletion carriers), ii) copy-number variant (CNV) outliers (i.e., individuals genotyped on plates with an average CNV count/plate >100 and individuals with >200 CNVs or a single CNV >10 Mb^19^), and iii) individuals with a duplication or non-manually validated deletion encompassed within chr16:28.6–29.2Mb. Among these, 59 unrelated (≤1^st^ degree) 16p11.2 breakpoint (BP)2–3 deletion carriers were retained (Figure 2). For all participants, self-reported gender and chromosomal sex were concordant. Participant characteristics are summarized in Table 1 and deletion calling procedure is described in 16p11.2 BP2-3 deletion detection *– UK Biobank*. Sensitivity analyses were carried out on a restricted set of 335,656 individuals of white British ancestry (in.white.British.ancestry.subset = 1 in ukb_sqc_v2.txt) which comprised 52 deletion carriers.

#### Matched cohort study

We aimed to identify 50 body mass index (BMI)-matched UKBB participants for each of the 59 deletion carriers (Figure 2). Matched participants were retained after excluding i) related UKBB participants (≤3^rd^ degree) and ii) individuals with 16p11.2 BP2-3 deletion. Participants were matched according to BMI (UK Biobank Field ID #21001; ±2.5 kg/m^2^), age (#21003; ±3.5 years), sex (#31; identical), and self-reported ethnic background (#21000; identical) without replacement (i.e., each control was used only once). We could not identify 50 matched participants for one deletion carrier of Bangladeshi ethnicity, who was therefore excluded. The final matched cohort analysis was performed on 58 deletion carriers and 2,900 matched control individuals. Participant characteristics are summarized in Table 1.

#### Estonian Biobank

The Estonian Biobank (EstBB) is a population-based cohort encompassing ∼20% of Estonia’s adult population, with 208,360 individuals (65% females) in the data freeze 2022v01 (2022-04-12).^31^ The activities of the EstBB are regulated by the Human Genes Research Act, which was adopted in 2000 specifically for the operations of the EstBB. Individual level data analysis in the EstBB was carried out under ethical approval 1.1–12/624 from the Estonian Committee on Bioethics and Human Research (Estonian Ministry of Social Affairs), using data according to release application 6–7/GI/2018 [2023/01/18] from the EstBB. All participants signed an informed consent form. Replication of association signals was carried out in a subset of 90,211 unrelated individuals of European ancestry after genotype/CNV quality control and pruning of related individuals (KING kinship coefficient >0.0884) and preferentially including i) deletion carriers and ii) individuals with phenotypic measurements. Among these, 19 deletion carriers were retained, with deletion calling procedure described in Detection of 16p11.2 BP2-3 deletion *– Estonian Biobank*.

### Method details

#### Detection of 16p11.2 BP2-3 deletion

##### UK biobank

Samples in the UKBB have been genotyped with either the Applied Biosystems UK Biobank Axiom Array, or the Applied Biosystems UK BiLEVE Axiom Array by Affymetrix, which share 95% probe overlap.^28^ We used two orthogonal approaches to identify high confidence 16p11.2 BP2-3 deletion carriers: fully automated CNV-calling and quality scoring pipeline and manual review of the genotyping fluorescence signal across the 16p11.2 BP1-5 region. Data was acquired in GRCh37/hg19 and unless specified otherwise, genomic coordinates follow this reference build.

We performed fully automated deletion detection and quality scoring, as previously described for genome-wide CNV detection in UKBB,^19^ to detect CNVs fully contained in chr16:28.6–29.2 Mb. This pipeline is based on PennCNV^29^ calls and offers the advantage of estimating breakpoints and assigning a probabilistic confidence quality score to each called deletion.^30^ To avoid using an arbitrary quality score cutoff to select deletion carriers, we manually reviewed fluorescent signal intensities (log R ratio, LRR) and B-allele frequency (BAF) in the 16p11.2 BP1-5 region (chr16:27-31 Mb) for the 272 deletion carriers identified through our automated pipeline, ensuring that each of them had a median probe-level LRR < −0.2 in 4 adjacent 16p11.2 BP2-3 regions covered by 20 probes (chr16:28,835,900-28,881,001; chr16:28,883,241-28,914,162; chr16:28,914,458-28,9668,35; chr16:28,970,852-29,001,460). We identified 60 unambiguous 16p11.2 BP2-3 deletion carriers (i.e., with no evidence of other CNV in the BP1-5 region). We established that 51 (85%) of these 60 individuals had a quality score ≤ −0.5 (i.e., stringent cutoff used for genome-wide studies) and all samples harboring a deletion with a quality score ≤ −0.5 were retained by manual review. This indicates that while an automated approach represents a good alternative when manual review is not feasible, the latter allows to boost power by retaining a few additional deletion carriers. The 60 identified deletion carriers included one pair of first-degree relatives (i.e., likely inherited deletion) of which the parent was excluded so that a total of 59 unrelated deletion carriers were taken forward for analyses (Figure S1; Table S1). Individuals identified as having a duplication or low confidence deletion (i.e., not validated by manual review) were excluded from the PheWAS (Figure 2).

##### Estonian Biobank

Illumina Global Screening Array (GSA) genotype data was available for 193,844 individuals included in the SNP imputation pipeline with matching genotype-phenotype identifiers and inferred versus reported sex, as well as an SNP-call rate ≥98%. Autosomal CNVs were called and quality-controlled as previously described, including exclusion of CNV outliers.^19^ Breakpoints of CNVs fully encompassed in chr16:28.8–29.1 Mb were visually inspected and retained if the distal coordinate of the deleted region encompassed or truncated *SH2B1* (chr16:28,857,921-28,885,526) and the proximal coordinate fully encompassed *LAT* (chr16:28,996,147-29,002,104). This resulted in 19 deletion carriers (63% females), among which 3 individuals had a fragmented deletion call.

#### Prevalence estimation of the 16p11.2 deletion

Prevalence of the 16p11.2 BP2-3 deletion in clinical and population cohorts was estimated based on literature review and data generated in this study (UKBB and EstBB estimates; Table S2). Prevalence in percentage was defined as the number of deletion carriers divided by the number of assessed individuals. To obtain estimates from the clinically ascertained DECIPHER database (https://www.deciphergenomics.org/; accessed 27/05/2022),^33^ we searched for CNVs affecting *SH2B1*, filtered for “Loss” to obtain deletions and retrieved 150 *SH2B1*-containing deletions. Deletions were further categorized according breakpoints by assigning the reported start and end of the deleted region to the closest segmental duplication obtained from UCSC segmental duplication track (accessed 06/07/2022; downloaded table: genomicSuperDups for chr16:21,000,000–34,800,000 (GRCh38), to match DECIPHER coordinates in GRCh38).^61^^,^^62^ Prevalence of the 16p11.2 BP2-3 deletion was calculated as a proportion of the total number of patients reported in DECIPHER (N ≈ 45,700).

#### Phenotype definition

##### Phenome-wide association scan

Hundred twelve traits, with an emphasis on metabolically relevant phenotypes, were selected for association study with the 16p11.2 BP2-3 deletion carrier status. For all traits, entries encoded as “do not know” or “prefer not to answer” were set as missing. Exact definitions of these traits and summary statistics are provided in Tables S3, S4, S5, and S6.

Thirty-three physical measurements were treated as continuous variables (ordinal traits were recoded as increasing continuous traits) and included 11 adiposity, 2 height, 7 childhood/puberty, 2 cardiovascular, 3 cognitive/behavioral, 5 physical activity, and 3 sleep-related measurements. Among these, 4 represent custom traits derived from existing data fields: systolic/diastolic blood pressures were inferred by completing *automated reading* (#4080/#4079) with *manual readings* (#93/#94) when the former was missing and waist-to-hip ratio (WHR) and WHR adjusted for BMI (WHRadjBMI) were calculated by dividing *waist circumference* (#48) by *hip circumference* (#49) and regressing out the effect of BMI and its interaction with sex for WHRadjBMI. We further assessed 58 blood biomarkers measured through blood biochemistry (N = 26), urine assays (N = 2), or nuclear magnetic resonance (NMR; N = 30). Among the latter, we included both raw and normalized (by *total fatty acids*; #23442) values for six fatty acid measurements. Continuous traits were inverse normal transformed before regressing out the effect of for sex, age, age^2^, genotyping batch and principal components (PCs) 1–40. For blood measurements, we further corrected for *fasting time* (#74), as well as fasting time squared if the latter parameter was significantly (p ≤ 0.01) impacting the trait when modeling $phenotype\;∼\;fasting\;time+{fasting\;time}^{2}$.

Twenty-one binary traits were evaluated, including 13 hospital diagnoses defined through International Classification of Diseases, 10^th^ Revision [ICD-10] codes, 4 self-reported mental health conditions, and self-reported usage of 4 medication classes. For ICD-10-based diagnoses, age at diagnosis was computed by subtracting matching *date at first in-patient diagnosis – ICD10* (#41280) from the birth date, calculated from the individual’s *month* (#52) and *year* (#34) *of birth* (birthday assumed on average to be the 15^th^). Results were converted in years by dividing by 365.25 to account for leap years.

##### Estonian Biobank replication

Association between 16p11.2 BP2-3 deletion carrier status and height, weight, and BMI were performed based on body measurements collected at recruitment. Traits were inverse normal transformed and corrected for sex, year of birth, genotyping batch (1–11) and PCs 1–20. Disease diagnoses are available as ICD-10 codes through crosslinking with national and hospital databases (last updated end 2021) and were used to replicate the association with diabetes, defined as any of the E10-E14 codes. Exact definitions and summary statistics are found in Tables S3 and S4.

##### Matched cohort study

Selected traits showing statistically significant or suggestive results in the PheWAS were followed up upon in our BMI-matched cohort study using curated phenotype definitions. Exact definitions and summary statistics are provided in Tables S7 and S8-46, respectively. Briefly, case definitions were obtained by combining ICD-10 codes (#41270) and information from self-reported diseases (#20002), disease-specific medication (#20003), and physical measurements or blood biomarkers at instance 0. Earliest documented age of onset was deduced from *date at first in-patient diagnosis – ICD10* (#41280), the age of onset of self-reported condition, or *age when attended assessment center* (#21003; instance 0) for physical measurements or blood biomarkers. Age at diagnosis was computed by adding the *age when attended assessment center* (#21003; instance 0) to the difference between the *date of attending assessment center* (#53; instance 0) and the date at diagnosis converted in years. Traits with no specific indication in Table S8 used the same definition as for the PheWAS.

#### Rare protein-coding variant burden tests

We used gene burden test results previously computed from 454,787 whole exome sequencing of the UKBB.^41^ Briefly, the study performed burden tests between ∼18,800 genes and ∼4,000 health-related traits using masks on variant function (i.e., predicted loss-of-function (pLoF)-only or pLoF and predicted deleterious missense variants) and minor allele frequency (MAF; i.e., MAF ≤1%, 0.01%, 0.001%, 0.0001%, or singletons). Association data with BMI and T2D (defined as E11 ICD-10 code) were extracted for the 9 genes in the 16p11.2 BP2-3 interval for all different test combinations and filtered for nominal significance (p ≤ 0.05).

#### Common variant associations at 16p11.2 BP2-3

##### GWAS catalog data

To determine whether common genetic variants in the 16p11.2. BP2-3 region had previously been found to impact traits we identified to be associated with the region’s deletion, we used the 16p11.2 BP2-3 coordinates ±50kb (chr16:28,811,314-29,035,178 in GRCh38)^63^ and retrieved all mapped associations from the GWAS Catalog (https://www.ebi.ac.uk/gwas/; accessed 22/12/2022).^42^ Coordinates of retrieved associations were converted to GRCh37 with the UCSC LiftOver tool (https://genome.ucsc.edu/cgi-bin/hgLiftOver) and involved traits were manually annotated with one of 12 trait categories.

##### Recombination rate estimation

Recombination rate was calculated by dividing the local difference in centimorgans (cM) by the local difference in Mb, using data from the HapMap^58^ lifted over to GRCh37 and downloaded from https://github.com/odelaneau/shapeit5/tree/main/maps.

#### Transcript Mendelian randomization

Transcriptome-wide Mendelian randomization (TWMR) was conducted following previously described methodology^44^^,^^60^ to determine whether changes in transcript levels of genes in the deleted 16p11.2 BP2-3 region causally modulate T2D risk. Exposures (i.e., transcript levels) were instrumented with independent genetic variants (r^2^ < 0.01), i.e., expression quantitative loci (eQTLs) for the gene of interest. Briefly, for the 6 genes with at least one eQTL (i.e., *ATXN2L*, *TUFM*, *SH2B1*, *AP2A1*, *NFATC2IP*, *SPNS1*), the effect of selected eQTLs on exposure (i.e., gene expression) and outcome (i.e., T2D risk) were used to estimate the causal effect of the former on the latter by inverse-weighted variance two-sample Mendelian randomization (Figure S4B). Genetic effect sizes on transcript levels (p ≤ 1 × 10-6) originate from either whole blood *cis*-eQTLs from the eQTLGen^45^ or tissue-specific *cis*-eQTLs from the GTEx project^46^ while those on T2D risk stem from a T2D genome-wide association study (GWAS).^59^ Prior to the analysis, datasets were harmonized and variants that are palindromic or had an allele frequency difference >0.05 between the datasets were removed.

#### Colocalization analysis

Genetic colocalization analysis was performed to determine whether genetically determined expression levels of the genes found to have a significant causal effect on T2D through TWMR (i.e., *TUFM*, *SH2B1*, *AP2A1*, *SPNS1*) shared a common genetic causal variant with the T2D GWAS signal. The same eQTL^45^ and GWAS^59^ summary statistics were used as in the TWMR analysis. Colocalization was performed with coloc.abf() from the R coloc package v5.1.0.1,^47^ using a 250kb window around the lead T2D GWAS signal (rs8046545; chr16:28,915,217; GRCh37) and following standard protocol.

### Quantification and statistical analysis

#### Phenome-wide association scan

Statistical analyses were performed in R v3.6.1.

Association between the 16p11.2 BP2-3 deletion carrier status (1 = deletion carrier; 0 = copy neutral; NA = duplication or non-manually validated deletion; see Detection of 16p11.2 BP2-3 deletion *– UK Biobank*) and normalized, covariate-corrected continuous traits (i.e., physical and blood measurements) were assessed through linear regression (lm()). For binary traits, logistic regressions (glm(family = binomial(link = “logit”))) were used to model the effect of deletion carrier status on disease/phenotype risk. As no correction for covariates was performed on binary traits, sex, age, age^2^, genotyping batch, and PC1-40 were included in the model. Model details are displayed in Tables S3, S4, S5, and S6.

##### Time-to-event analysis

To assess whether 16p11.2 BP2-3 deletion carrier status also influenced the age of onset of ICD-10-based diseases we used Cox proportional-hazard models implemented in the survival R package.^64^ For this purpose, we used the earliest documented disease onset (see phenotype definition – *Phenome-wide association scan*) for cases and the date of the last reported diagnosis across all individuals (30/09/2021) minus the birth date converted in years for controls. Sex, age, age^2^, genotyping batch, and PC1-40 were included in the regression model (Table S4).

##### Multiple testing correction

Some of the 112 assessed traits are highly correlated and thus not independent. We accounted for this in our multiple testing strategy by calculating the number of effective tests, i.e., the number of tests required to explain 99.5% of the variance in the phenotypic dataset.^65^ This number was estimated to 88, both when considering all ancestries or only the subset of white British individuals, setting the strict threshold for genome-wide significance at p ≤ 0.05/88 = 4.7 × 10^−4^ for the PheWAS. Nominal significance refers to p ≤ 0.05.

#### Replication in the Estonian Biobank

Association between the 16p11.2 BP2-3 deletion carrier status and normalized, covariate-corrected continuous traits (i.e., BMI, weight, height) and binary outcomes (i.e., diabetes) were performed using linear and logistic regressions, respectively, following the same procedure as described for UKBB (see Phenome-wide association scan). Sex, year of birth, genotyping batch (1–11), and PC1-20 were included as covariates for the association with diabetes (Table S3). As all replicated signals were concordant in direction, we reported one-sided p values, which were deemed significant at p ≤ 0.05/4 = 0.0125 to account for the 4 performed tests.

#### Matched cohort study

Statistical analyses were performed in R v4.1.1. Detailed methodology including covariates, statistical tests and results are reported for each trait in the main text or in Tables S8 and S9.

##### Trait analysis

For continuous traits, linear models were implemented with lm() and cohens_f() from the package effect size v0.8.2 were used to estimate effect sizes. We considered the main effect (i.e., effect of the deletion compared to matched non-carriers as a baseline) and interactions with relevant covariates (e.g., lipid lowering drug, when assessing cholesterol levels). If continuous traits were not normally distributed, Wilcoxon rank-sum was applied (wilcox.test()) and effect sizes were estimated with rFromWilcox().^66^ All *post-hoc* analyses were performed using the Tukey’s procedure from the lsmeans package v2.30-0^67^^,^^68^ ‘lsmeans’ R package, vers. 2.30–0; for the respective interactions assessed in linear models. Nominal traits were assessed with logistic regression (glm(family = binomial(link = “logit”))) or with Fisher’s exact test (fisher.test()) for which effect sizes were estimated as odds ratios (OR).

##### Time-to-event analysis

Association between deletion carrier status and age at condition onset were implemented as previously described in phenome-wide association scan – *Time-to-event analysis*. We used the earliest documented age at disease onset (see phenotype definition *– Matched cohort study*) for cases and the last documented age without diagnoses otherwise. To determine the latter, *age when attended assessment center* (#21003; instance 0; for physical measurements or blood biomarkers) and age of last documented ICD-10 diagnosis were considered. The age of the last documented ICD-10 diagnosis was determined by subtracting the *date of attending assessment center* (#53; instance 0) from the last date of all *date at first in-patient diagnosis – ICD10* (#41280), converting the result in years by dividing trough 365.25 to account for leap years and adding it to the *age when attended assessment center* (#21003; instance 0). Of the *age when attended assessment center* (#21003; instance 0), and the age of the last documented ICD-10 diagnosis, the oldest age was defined as last documented age without diagnosis. Results were plotted with Kaplan-Meier curves.

##### Multiple testing correction

Reported p values are nominal and two-sided. Bonferroni threshold for testing ∼40 traits is 0.05/40 = 0.00125.

#### Transcriptome-wide Mendelian randomization

TWMR estimates were considered significant when p ≤ 0.05/9 = 5.6 × 10^−3^ to account for the nine genes in the 16p11.2 BP2-3 interval. We used standardized genetic effect sizes, therefore TWMR estimates can be interpreted as the phenotypic impact of one standard deviation increase in expression. Since we expect the deletion to decrease expression, negative TWMR effects (i.e., increased expression decreases T2D risk) were considered directionally concordant with the association study results (i.e., deletion increase T2D risk).

#### Colocalization analysis

For each tested gene, coloc outputs the posterior probability supporting 5 different scenarios.^47^ Evidence for shared causal genetic signal from the eQTL and GWAS data (i.e., scenario H_4_) was considered when the posterior probability for that hypothesis was PP_H4 > 0.8.

## Data Availability

• This paper analyzes existing, publicly available data. The accession numbers for the datasets are listed in the key resources table. • Statistical tests and published code are listed in the STAR Methods and key resources table. • Any additional information required to reanalyze the data reported in this work paper is available from the lead contact upon request.

## Supplemental information

Document S1. Figures S1–S4

Table S1. Breakpoint and quality score of the 59 UKBB 16p11.2 BP2–3 deletion carriers, related to Figures 1 and 2 Start and end coordinates of deletion breakpoints (GRCh37/hg19) for the 59 deletion carriers considered in the PheWAS. We report the copy number, length in base pairs and microarray probes, and quality score of each deletion.

Table S2. Prevalence of the 16p11.2 BP2–3 deletion in clinical and population cohorts, related to Figures 1 and 2 Estimation of the prevalence of the 16p11.2 BP2–3 deletion from the literature and this study. The cohort type distinguishes between clinical versus population-based cohorts, with the cohort’s name, publication in which deletion prevalence was estimated, and specific ascertainment bias indicated. For each cohort, the sample size and number of 16p11.2 BP2-3 deletion carriers is indicated and was used to infer prevalence. Cohorts were aggregated by cohort type to get the average prevalence in clinical versus population-based cohorts. The last two rows indicate the prevalence in a meta-analysis of 17 cohorts. We report the 16p11.2 BP2–3 deletion prevalence among individuals with one or more versus none of the disease phenotypes assessed by the study.

Table S3. Summary statistics of the 16p11.2 BP2–3 deletion PheWAS, physical measurements, related to Figures 3, 4, and 5 Physical measurements assessed in the in PheWAS analysis. For each trait, we indicate the UKBB field ID (“/” = phenotypes are divided by each other; “adj.” = corrected for the indicated phenotype; “|” = either phenotype value that was available), description, unit of measurement, and broad trait category. When available, we report trait summary values (count, mean, SD, median, minimum, and maximum) for copy-neutral (blue) and deletion carriers (red) when considering all UKBB individuals (UKBB, all ancestries), only white British individuals (UKBB, white British), or the EstBB replication cohort (EstBB). Summary statistics (effects size in standard deviations (beta [SD]), standard error (SE), and p value) of the linear regression testing for an association between deletion carrier status and trait are reported for each cohort (purple).

Table S4. Summary statistics of the 16p11.2 BP2–3 deletion PheWAS ICD-10 codes, related to Figures 3, 4, and 5 Description of the ICD-10-based disease diagnoses assessed in the PheWAS analysis. For each disease, we indicate the considered codes (including all subcodes), the disease’s description and broad category, as well as some additional comments related to the case-control definition. When available, we report trait summary values (number of cases, mean, SD, and median age at onset in years) for copy-neutral (blue) and deletion carriers (red) when considering all UKBB individuals (UKBB, all ancestries), only white British individuals (UKBB, white British), or the EstBB replication cohort (EstBB). Summary statistics of the logistic regression testing for an association between the deletion carrier status and disease risk (effects size [beta], SE, OR, and p value) and Cox proportional-hazards testing for an association with disease age at onset (coefficient and p value) are reported for each population (purple).

Table S5. Summary statistics of the 16p11.2 BP2–3 deletion PheWAS, other binary traits, related to Figures 3, 4, and 5 Description of binary traits assessed in the PheWAS analysis. For each trait, we indicate the UKBB field ID (“|” = combine data from both fields), description, encoding of cases (either the encoding in the respective field ID or the considered codes), and broad trait category. We report the number of cases for copy-neutral (blue) and deletion carriers (red) when considering all UKBB individuals (UKBB, all ancestries) or only white British individuals (UKBB, white British). Summary statistics of the logistic regression testing for an association between the deletion carrier status and case status (effects size [beta], SE, OR, and p value) are reported for each population (purple).

Table S6. Summary statistics of the 16p11.2 BP2–3 deletion PheWAS, blood measurements, related to Figures 3, 4, and 5 Description of the blood measurements assessed in the PheWAS analysis. For each trait, we indicate the UKBB field ID (“/” = phenotypes are divided by each other), description, unit of measurement, and broad trait category. When available, we report trait summary values (count, mean, SD, median, minimum, and maximum) for copy-neutral (blue) and deletion carriers (red) when considering all UKBB individuals (UKBB, all ancestries) or only white British individuals (UKBB, white British). Summary statistics (effects size in beta [SD], SE and p value) of the linear regression testing for an association between deletion carrier status and trait are reported for each population (purple). Columns “FT” and “FT2” indicate whether fasting time (#74) and fasting time squared, respectively, were included (= 1) or not (= 0) as covariates in the linear regression model.

Table S7. Disease definitions for matched cohort analyses, related to Figures 4 and 5 Refined disease definitions for the BMI-matched cohort analyses. For each trait, we provide the conditions under which the listed UKBB field ID (“|” = combine data from both fields) are considered as cases if individuals meet the criteria under “value” for the given trait. Similarly, for disease phenotypes, the age at onset was defined as the earliest documented occurrence in the listed field IDs.

Table S8. Summary statistics for the matched cohort analyses, related to Figures 4 and 5 The UKBB field ID (“|” = combine data from both fields) or table in which the assessed traits are defined are listed in the first column. The descriptive statistics provides the mean ± SEM for continuous traits, the prevalence for binary traits, and the mean age at onset ±SEM for binary traits assessed through Cox proportional-hazard model for both deletion carriers and matched participants. Inferential statistics of the comparison between deletion carriers and matched controls are reported, including the p values, test statistics (Wilcoxon test, W; other tests, detailed test statistics provided in indicated tables) and effect sizes (Wilcoxon test, r; Cox proportional-hazards model, HR with 95% confidence interval [CI]; other tests, detailed effect sizes provided in indicated tables).

Table S9. Statistical tests for the matched cohort analyses, related to STAR Methods Reported p values are nominal and two-sided (∗p < 0.05, ∗∗p < 0.01, ∗∗∗p < 0.001).

Table S10. Gene burden tests between BMI, plasma lipids, and T2D for the genes encompassed in 16p11.2 BP2–3, related to STAR Methods Results from the rare variant gene burden tests performed by Backman et al.^41^ between the nine genes encompassed in the 16p11.2 BP2–3 interval and BMI, plasma lipids (total cholesterol, direct LDL, HDL, and triglyceride levels), and T2D defined through the E11 ICD-10 code. Tests were performed using different masks regarding the function (predicted loss of function [pLoF] only or pLoF and predicted deleterious missense variants) and MAF of the considered variants. We report the frequency of the effect allele (EA), the estimated effect size (beta for BMI and plasma lipids; OR for T2D), and p value. Filter is set to display only nominally significant associations (p ≤ 0.05).

Table S11. GWAS catalog SNP-GWAS lead signals for the 16p11.2 BP2–3 region, related to STAR Methods Listed associations on the GWAS catalog for the 16p11.2 BP2–3 ±50 kb region. For each signal, the lead risk allele is provided along with its position in GRCh38/hg38 (reported in the GWAS catalog) and GRCh37/hg19 (lifted over). Summary statistics of the reported associations include, when available, p value, specific annotations, risk allele frequency (RAF), OR, beta (effect size) in corresponding unit, 95% CI, and mapped gene. Based on the report trait, we manually assigned each signal to a broad category (“trait category”). GWAS catalog accession number, PubMed identifier, and name of the first author of the study reporting the association are listed.

Table S12. Transcript Mendelian randomization estimating the causal effect of changes in the expression of 16p11.2 BP2–3 genes on T2D risk in whole blood (eQTLGen), related to STAR Methods Transcript MR was used to assess the causal effect of changes in the expression of six of the nine genes on 16p11.2 BP2–3 with at least one eQTL for T2D risk in the whole-blood eQTLGen dataset. For each gene, the ENSEMBL identifier is reported, along with the number of eQTLs used to instrument the MR. Identity (top eQTL), position in GRCh37/hg19 (top eQTL position), effect (top eQTL A1), and alternative allele (top eQTL A2) of the strongest eQTLs are reported. Inverse-weighted variance MR effect size (MR effect), SE, and p value are indicated.

Table S13. Tissue-specific transcript Mendelian randomization estimating the causal effect of changes in the expression of 16p11.2 BP2–3 genes on T2D (GTEx), related to STAR Methods Tissue-specific transcript MR was used to assess the causal effect of changes in the expression of six of the nine genes on 16p11.2 BP2–3 with at least one eQTL for T2D risk in at least on tissue of the Genotype-Tissue Expression (GTEx) project. For each gene, the ENSEMBL identifier is reported, along with the number of eQTLs used to instrument the MR. Identity (top eQTL) of the strongest eQTLs are reported. Inverse-weighted variance MR effect size (MR effect) and p value are indicated.

Table S14. Colocalization analysis between the expression of 16p11.2 BP2–3 genes and T2D GWAS signal, related to STAR Methods Colocalization analysis was performed for four of the nine genes on 16p11.2 BP2–3 with (1) at least one eQTL and (2) a significant Mendelian randomization effect between transcript levels and T2D risk. For each gene, the ENSEMBL identifier is reported, along with the number of variants considered for the analysis (±250 kb from the lead T2D variant). The PP is given for the scenario in which (1) neither trait has a genetic association in the region (PP_H0); (2) only the eQTL has a genetic association in the region (PP_H1); (3) only T2D GWAS has a genetic association in the region (PP_H2); (4) both traits are associated, but with different causal variants (PP_H3); (5) and both traits are associated and share a single causal variant (PP_H4).

Document S2. Article plus supplemental information
