# Supplementary material for: Chromosomal deletions on 16p11.2 encompassing SH2B1 are associated with accelerated metabolic disease
Source: Cell Rep Med. 2023 Aug 15;4(8):101155. doi: 10.1016/j.xcrm.2023.101155 (PMC10439272; doi:10.1016/j.xcrm.2023.101155)
Supplement: Document S1. Figures S1–S4 [file mmc1.pdf]

Cell Reports Medicine, Volume *4*

Supplemental information

Chromosomal deletions on 16p11.2 encompassing *SH2B1* are associated with accelerated metabolic disease

Ruth Hanssen, Chiara Auwerx, Maarja Jõeloo, Marie C. Sadler, Estonian Biobank Research Team, Elana Henning, Julia Keogh, Rebecca Bounds, Miriam Smith, Helen V. Firth, Zoltán Kutalik, I. Sadaf Farooqi, Alexandre Reymond, and Katherine Lawler

CONTENTS

Supplemental Figures S1-4

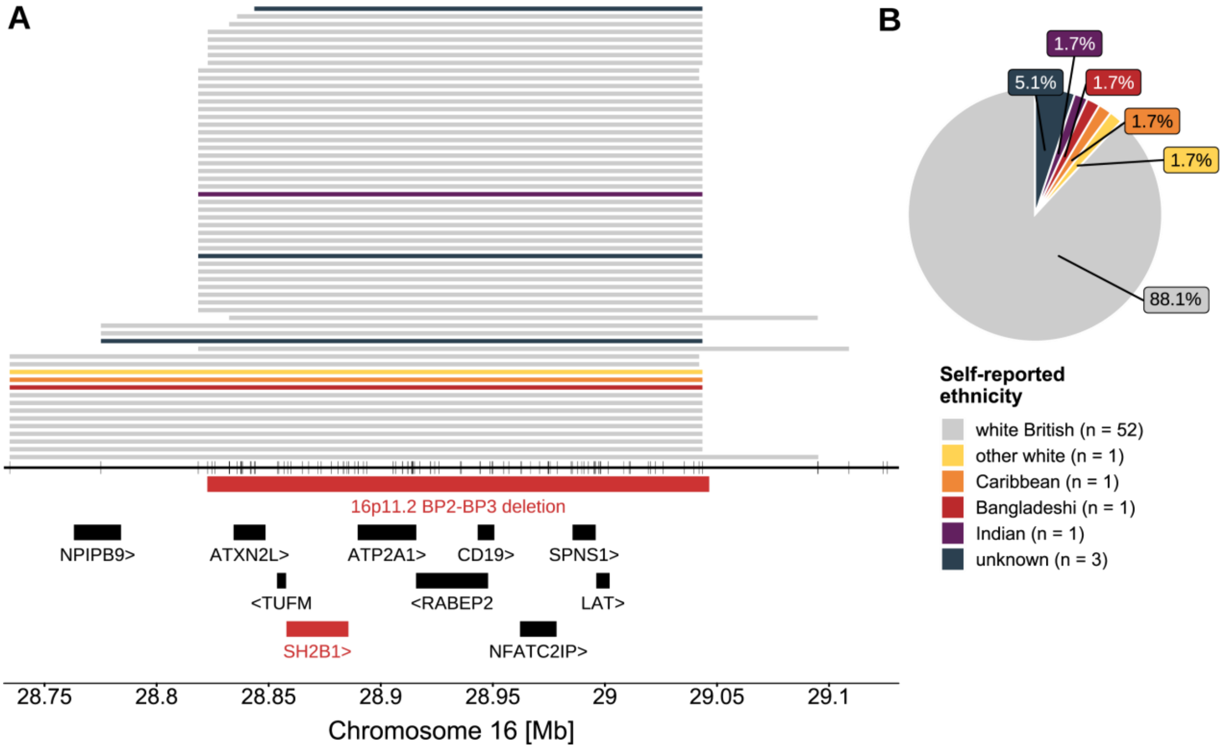

Figure S1. Characteristics of 16p11.2 BP2-3 deletion carriers in UK Biobank. Related to Figure 2 and Table 1. (A) Breakpoints of the 59 unrelated 16p11.2 BP2-3 deletion carriers included in the phenome-wide association scan (PheWAS) determined through an automated CNV calling pipeline. Each line represents one individual according to self-reported ethnic background (legend in **B**). Vertical ticks indicate the location of genotyping probes on the microarray from which deletions were called (middle). Genomic location and orientation of the recurrently deleted BP2-3 region including *SH2B1* in red, along with other genes in the region in black. (B) Percentage of 59 unrelated deletion carriers belonging to each ethnic group; sample size indicated in the legend (n).

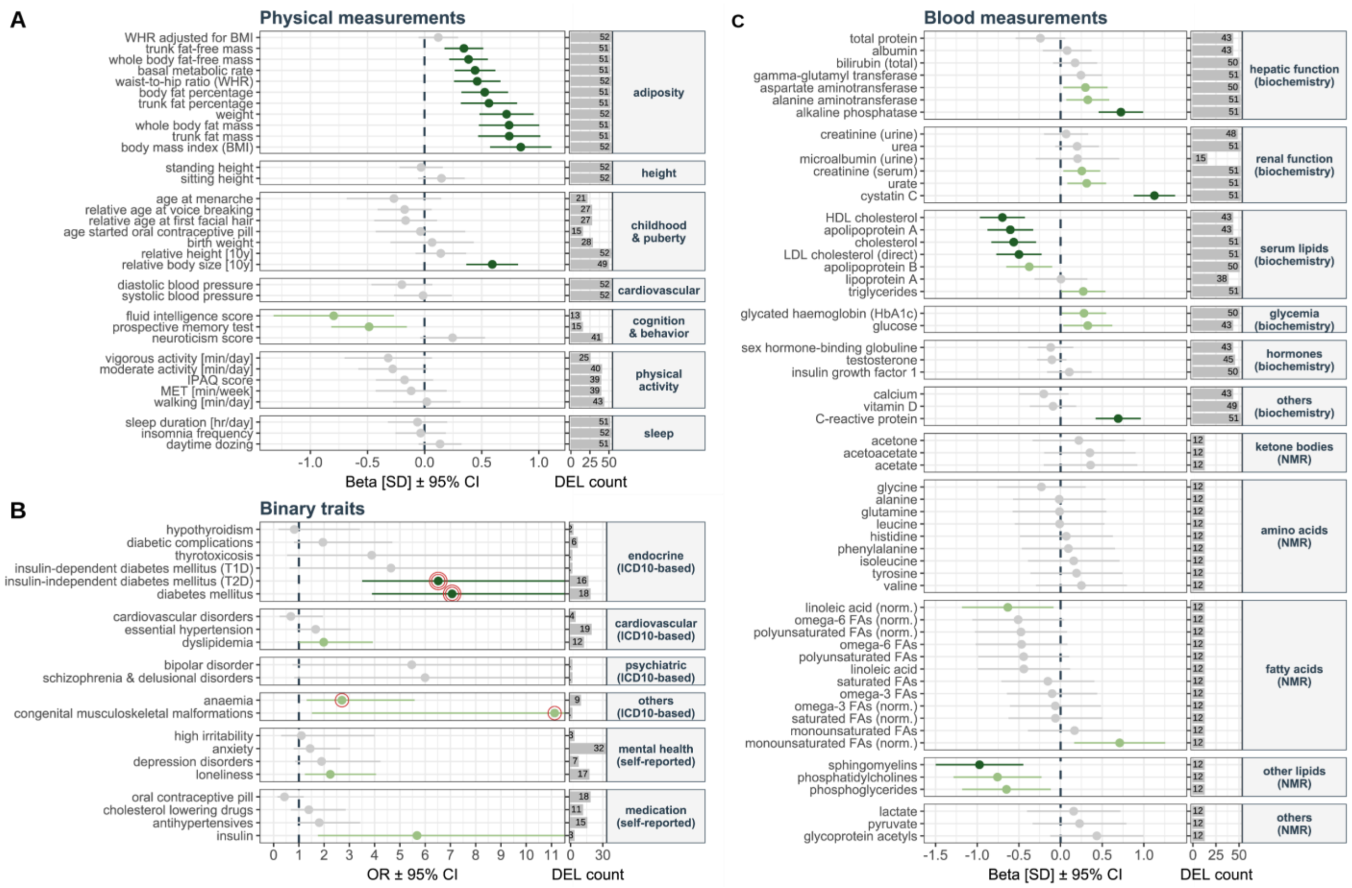

Figure S2. Sensitivity phenome-wide association scan in participants of white British ancestry. Related to Figure 3. Results of the Phenome-Wide Association Scan (PheWAS) for (A) 33 physical measurements, (B) 21 binary traits, and (C) 58 blood measurements according to trait category (y-axis). (A, C) Left panel, x-axis shows the effect of the deletion (beta) on each trait in standard deviations (SD) with error bars representing 95% confidence intervals (CI). (B) Left panel, x-axis shows the odds ratio (OR) with error bars representing the 95% CI. Upper range of the CI truncated for some traits to facilitate visualization. Color indicates level of statistical significance, dark green ($p \leq 0.05/88 = 4.7 \times 10^{-4}$), light green ($p \leq 0.05$) and grey (non-significant). ICD-10 based diagnoses were assessed with a Cox proportional-hazards model and strictly ($p \leq 0.05/88 = 4.7 \times 10^{-4}$) and nominally ($p \leq 0.05$) significant associations between deletion carrier status and early onset of the disease are indicated by a double or single red circle surrounding the OR, respectively. The vertical dashed line represents a null effect size. Right panel, x-axis indicates the number of deletion carriers (DEL, maximum = 52) in whom the trait was measured (A, C) or the number of cases for the considered trait (B).

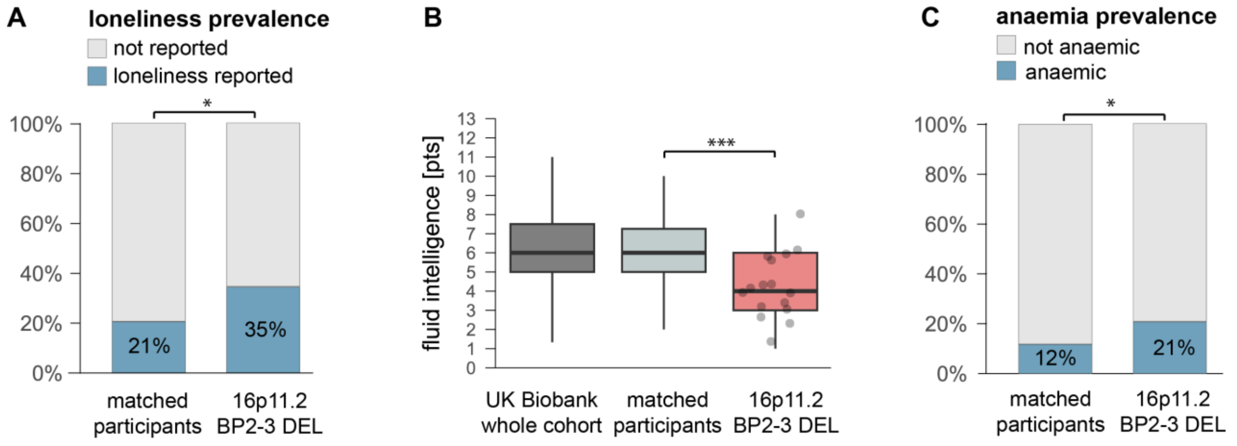

Figure S3. 16p11.2 BP2-3 deletion carriers have cognitive impairment. Related to Figure 3.
(A) Prevalence [%] of self-reported loneliness among deletion carriers (16p11.2 BP2-3 DEL) and BMI matched participants. * = $p < 0.05$. (B) Fluid intelligence score [points] on a scale from 0 to 13 in control individuals from the phenome-wide association scan (PheWAS; UK Biobank whole cohort; dark grey), matched participants (light grey) and deletion carriers (16p11.2 BP2-3 DEL; red). Data points are depicted only for deletion carriers (N=16 with available fluid intelligence score). *** = $p < 0.001$. (C) Prevalence [%] of anaemia among matched participants and deletion carriers (16p11.2 BP2-3 DEL). * = $p < 0.05$ for the matched cohort analysis.

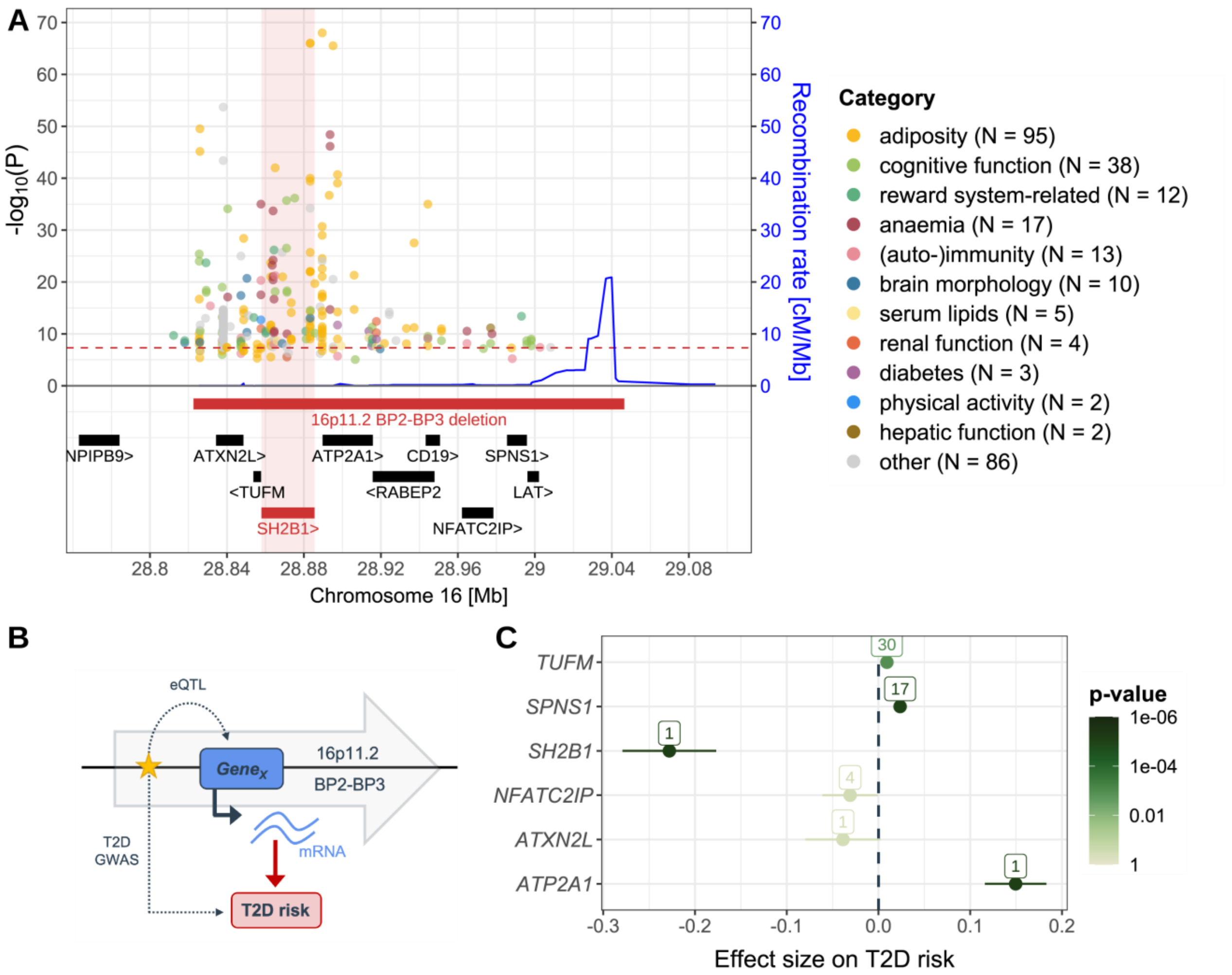

Figure S4. Common variant associations and transcriptome-wide Mendelian randomization effects at the 16p11.2 BP2-3 region. Related to STAR Methods, Method details

(A) Single-nucleotide polymorphism (SNP)-genome-wide association study (GWAS) signals retrieved from the GWAS Catalog for the 16p11.2 BP2-3 region ± 50 kb. The x-axis represents the genomic coordinates (GRCh37). Top: Left y-axis indicates the negative logarithm of reported association p-values, with each signal coloured according to a manually assigned broader trait category. Number of signals per category is indicated (N). Right y-axis indicates the local recombination rate in cM/Mb and is represented as a blue line. The dashed horizontal red line indicates the commonly accepted threshold for GWAS genome-wide significance at $p \leq 5 \times 10^{-8}$. Bottom: Genomic location and orientation of the recurrently deleted region and *SH2B1* in red, along with other genes in the region in black.

(B) Schematic representation of the transcriptome-wide Mendelian randomization (TWMR) approach that was applied to six 16p11.2 BP2-3 genes with at least one expression quantitative trait locus (eQTL). First, eQTLGen data from Võsa et al., 2021 was used to identify independent *cis*-eQTLs (yellow star) for the assessed gene (blue box) and the effect of these variants on the expression of the gene was retrieved (dotted arrow labeled “eQTL”). Next, the effect of the same variants on type 2 diabetes (T2D) risk was assessed based on T2D genome-wide association study (GWAS) summary statistics from Mahajan et al., 2018 (dotted arrow labeled “T2D GWAS”). These quantities were used to estimate the causal impact of one standard deviation increase in the expression of the assessed gene on T2D risk (red arrow) based on inverse-weighted variance two-sample Mendelian randomization.

(C) Transcriptome-wide Mendelian randomization (TWMR) estimates with standard error (x-axis) representing the causal effect of changes in expression of six 16p11.2 BP2-3 genes with at least one expression quantitative trait locus (eQTL; y-axis) on type 2 diabetes (T2D) risk. Estimates are colored according to the p-value, with the threshold for significance at $p \leq 0.05/9 = 5.6 \times 10^{-3}$. Labels indicate the number of eQTLs used to estimate TWMR effects.
